# Repurposing FDA-Approved Drugs as Nav1.7 Channel Modulators: An Integrated Structure-Based Virtual Screening and Molecular Dynamics Study

**DOI:** 10.3390/ijms27146476

**Published:** 2026-07-21

**Authors:** Mena Abdelsayed, Yassir Boulaamane

**Affiliations:** 1 Lankenau Institute for Medical Research, Philadelphia, PA 19096, USA; 2 Laboratory of Innovative Technologies, National School of Applied Sciences of Tangier, Abdelmalek Essaadi University, Tetouan 93000, Morocco

**Keywords:** Nav1.7, drug repurposing, structure-based virtual screening, molecular dynamics simulations, FDA-approved drugs, non-opioid analgesics

## Abstract

The voltage-gated sodium channel Nav1.7 is a strongly validated target for the development of novel, non-opioid analgesics due to its genetic link to pain signaling. To accelerate the discovery of safe Nav1.7 modulators, this study outlines an integrated computational pipeline to repurpose FDA-approved drugs. A structurally complete model of the Nav1.7 central pore was generated via homology modeling from a high-resolution cryo-EM structure (PDB: 7W9K) to ensure a physically consistent model suitable for dynamic simulations. We conducted a structure-based virtual screening of 2296 FDA-approved compounds, identifying four promising candidates (DB04868, DB00941, DB01419, and DB15982) with strong predicted affinities ranging from −11.38 to −12.57 kcal/mol. Interaction fingerprinting revealed that binding is predominantly driven by hydrophobic contacts with conserved pore-lining residues, including Phe1503, Leu1010, and Ile1500. To validate these static predictions, the top protein–ligand complexes were subjected to single-replica 250 ns molecular dynamics (MD) simulations. Comprehensive trajectory analyses, including RMSD, RMSF, and principal component analysis, revealed a notable discrepancy between static docking scores and dynamic stability. The highest-scoring docking candidate, DB04868, exhibited substantial conformational flexibility and reduced stabilization under simulated physiological conditions. Conversely, DB01419, despite a lower initial docking rank, demonstrated the highest structural stability across all metrics and uniquely formed intermittent stabilizing hydrogen bonds. These findings underscore the value of post-docking MD validation in computational drug discovery and nominate DB01419 and DB15982 as candidate scaffolds that warrant subsequent experimental validation, including electrophysiological characterization and Nav-isoform selectivity profiling. We emphasize that these are computational predictions: in silico binding stability is not equivalent to functional inhibition of Nav1.7 currents, and the lead designations reported here remain hypothesis-generating until confirmed by patch-clamp and biochemical assays.

## 1. Introduction

The urgent need for effective, non-addictive treatments for chronic pain has driven a paradigm shift in analgesic drug discovery, with the voltage-gated sodium channel Nav1.7 emerging as a premier therapeutic target. The validation for Nav1.7 is exceptionally strong, underpinned by compelling human genetic evidence: gain-of-function mutations in the *SCN9A* gene are linked to severe pain syndromes such as inherited erythromelalgia, whereas loss-of-function mutations result in congenital insensitivity to pain [1,2]. Furthermore, the preferential expression of Nav1.7 in the peripheral nervous system, specifically within dorsal root ganglion and sympathetic neurons, positions it as an ideal target for non-opioid analgesics with potentially superior safety margins compared to globally active sodium channel blockers [3,4].

Despite this overwhelming biological rationale, translating Nav1.7 biology into clinical therapeutics has proven remarkably challenging. A well-established strategy involves targeting the channel’s central pore cavity, a highly hydrophobic region known to accommodate various pore-blocking drugs, including local anesthetics and antiepileptics [5,6]. However, the high sequence conservation of this domain across different sodium channel (Nav) isoforms presents a formidable hurdle for achieving subtype selectivity [7]. To overcome these developmental bottlenecks, drug repurposing presents a highly strategic advantage. By screening libraries of FDA-approved compounds, researchers can leverage pre-established safety, pharmacokinetic, and regulatory profiles to substantially accelerate the translational timeline of novel pain therapeutics [8].

Computationally, structure-based virtual screening is a cornerstone of modern drug repurposing; yet, relying solely on static molecular docking poses significant risks. Static docking algorithms often fail to account for receptor flexibility and induced-fit phenomena, leading to a well-documented disconnect between predicted binding scores and actual dynamic stability within the physiological environment [9]. For a highly dynamic membrane protein like Nav1.7, identifying ligands that can maintain stable interactions—particularly by balancing hydrophobic complementarity with transient polar contacts—requires a more rigorous, dynamic approach. Consequently, post-docking validation utilizing extensive molecular dynamics (MD) simulations has become an indispensable step in prioritizing robust computational hits [10].

In this study, we present an integrated, high-throughput computational pipeline aimed at identifying novel Nav1.7 channel modulators from existing pharmacopoeia. The workflow was deliberately structured as a hierarchical funnel, motivated by two considerations. First, although docking can rapidly triage thousands of approved drugs, its scoring functions are known to correlate only weakly with true binding free energies and to neglect receptor flexibility and solvent reorganisation, so docking is used here only as an enrichment filter rather than as the basis for final ranking. Second, because the Nav1.7 pore is a hydrophobic, conformationally dynamic cavity, candidates that appear optimal in a single static pose may not survive realistic thermal motion; we therefore added post-docking molecular dynamics as an orthogonal, physics-based filter to test whether each top hit retains its pose and stabilises the channel over hundreds of nanoseconds. This static-to-dynamic design directly motivates the central comparison reported below. Utilizing a complete, high-quality homology model derived from the 2.2 Å cryo-EM structure of Nav1.7 [11], we performed a structure-based virtual screening of 2296 FDA-approved drugs targeting the central pore using AutoDock Vina (v1.2.6) [12]. Crucially, to bridge the gap between static predictions and dynamic reality, the top-ranked candidates were subjected to single-replica 250 ns MD simulations. By systematically evaluating backbone deviations, ligand persistence, and interaction fingerprints, this study identifies candidate scaffolds for downstream experimental characterization and illustrates the value of integrating multi-parameter MD analyses with docking-based prioritization, given the well-documented limitations of static docking scores as predictors of dynamic binding stability.

## 2. Results

### 2.1. Molecular Docking Results

**Figure 1:**
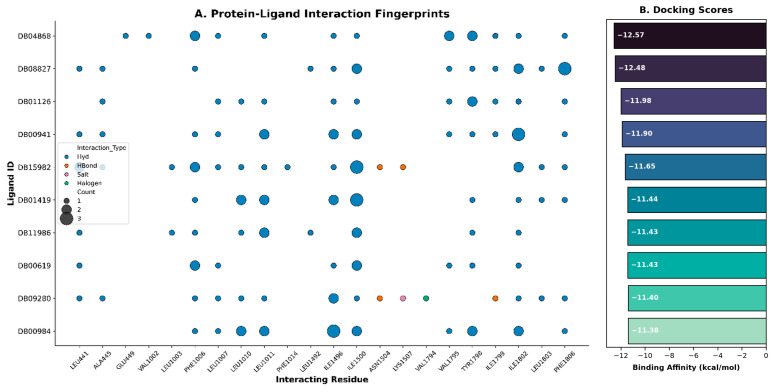
Docking scores and protein–ligand interaction fingerprints of top-ranked FDA-approved compounds targeting the Nav1.7 pore. (**A**) Interaction fingerprint matrix mapping Ligand IDs against interacting residues. Marker colors indicate interaction types: blue for hydrophobic (Hyd), orange for hydrogen bonds (HBond), pink for salt bridges (Salt), and green for halogen bonds (Halogen). Marker size represents interaction count, scaling from 1 (smallest) to 3 (largest). (**B**) Horizontal bar chart of predicted binding affinities (kcal/mol), with exact scores printed inside. The color gradient transitions from dark purple/black (strongest affinity, −12.57 kcal/mol) to light mint green (weakest affinity, −11.38 kcal/mol).

Structure-based virtual screening of FDA-approved compounds against the Nav1.7 pore identified several candidates with strong predicted binding affinities, ranging from −12.57 to −11.38 kcal/mol (Figure 1). These values are comparable to or exceed scores reported in previous AutoDock Vina-based screens targeting Nav1.7 [12,13]. DB04868 showed the highest affinity (−12.57 kcal/mol), followed by DB08827 and DB01126, while DB00941, DB15982, and DB01419 consistently scored below −11.5 kcal/mol. For translational clarity, the four compounds advanced to molecular dynamics correspond to nilotinib (DB04868; a BCR-ABL tyrosine-kinase inhibitor indicated for chronic myeloid leukaemia), hexafluronium (DB00941; a neuromuscular-blocking adjunct used in anaesthesia), antrafenine (DB01419; an analgesic/anti-inflammatory piperazine derivative), and berotralstat (DB15982; a plasma kallikrein inhibitor used for hereditary-angioedema prophylaxis); the two higher-scoring compounds that were not advanced, DB08827 and DB01126, correspond to lomitapide (a microsomal triglyceride-transfer-protein inhibitor) and dutasteride (a 5-alpha-reductase inhibitor), respectively. These original indications are unrelated to sodium-channel pharmacology and are reported here solely to document the repurposing provenance of the hits; none implies any previously established Nav1.7 activity.

Interaction fingerprint analysis indicated that binding is primarily driven by hydrophobic contacts within the pore, involving key residues such as Phe1503, Leu1010, and Ile1500, complemented by hydrogen bonding with residues including Asn1504 and Ser1004 [5,14]. Compounds such as DB00941 and DB01419 displayed interaction-rich profiles, whereas DB15982 exhibited a more diverse interaction pattern, incorporating both polar and occasional ionic contacts.

**Figure 2:**
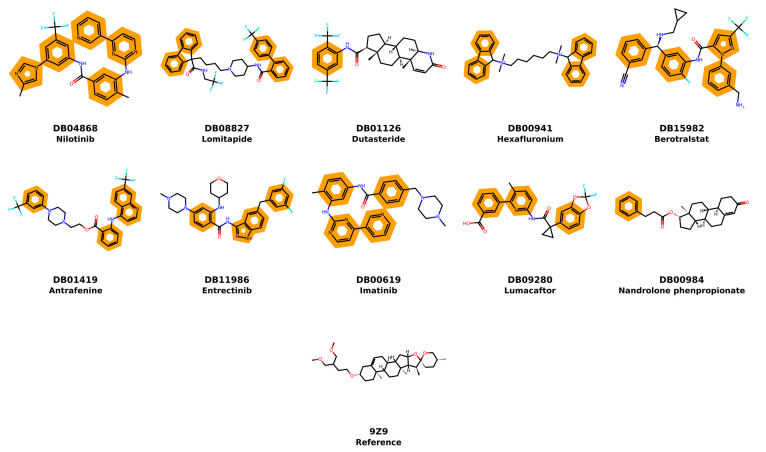
Chemical structures of the top-ranked compounds and the reference ligand (9Z9).

The top-ranked compounds (Figure 2) span diverse chemical classes but share common features, notably extended aromatic systems and lipophilic scaffolds, consistent with the hydrophobic nature of the Nav1.7 pore [5,6]. The reference ligand (9Z9) similarly exhibits a predominantly hydrophobic character, reinforcing the importance of van der Waals interactions for binding. A comparable profile is observed for DB00984 (Nandrolone phenylpropionate); however, despite its structural similarity and favourable lipophilicity, such compounds are known to suffer from poor selectivity and suboptimal pharmacokinetics, highlighting that hydrophobicity alone is insufficient for successful drug development.

**Figure 3:**
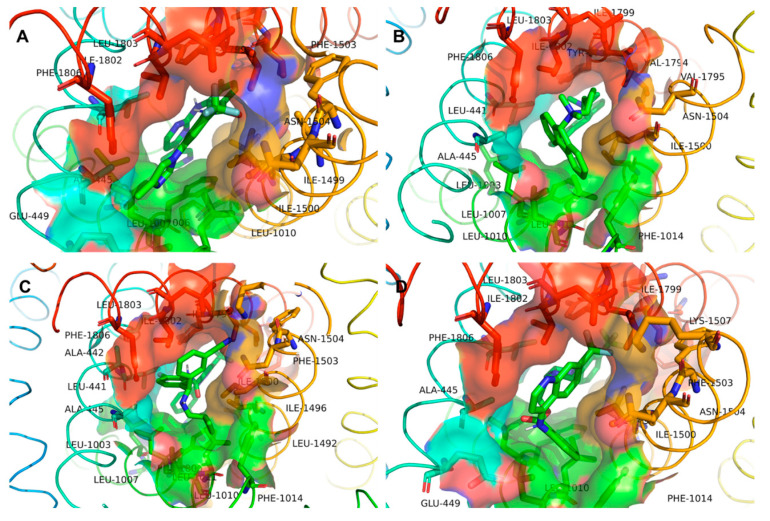
Predicted binding poses of the four compounds selected for molecular dynamics simulations within the Nav1.7 central pore: (**A**) DB04868, (**B**) DB00941, (**C**) DB15982, and (**D**) DB01419. Each panel shows the ligand positioned in the pore cavity relative to key pore-lining residues. Dashed lines, where present, indicate predicted polar contacts such as hydrogen bonds; hydrophobic contacts are inferred from close ligand–residue proximity within the binding pocket. The poses illustrate that all four compounds occupy a similar pore region, but differ in interaction patterns: DB04868 adopts a deeply buried hydrophobic pose, DB00941 and DB01419 show additional stabilising contacts, and DB15982 displays a more flexible interaction profile.

Binding mode analysis (Figure 3) shows that all selected compounds occupy a similar region within the central cavity. DB04868 adopts a deeply buried, well-defined pose dominated by hydrophobic interactions. DB00941 and DB01419 form multiple stabilising contacts, including polar interactions with Asn1504, while DB15982 demonstrates a flexible binding mode with a diverse interaction profile. Collectively, these results highlight the importance of balancing hydrophobic complementarity with polar interactions to achieve stable and potentially selective binding within the Nav1.7 pore [14,15].

### 2.2. Molecular Dynamics Trajectory Analysis

Molecular dynamics simulations were performed to evaluate the structural stability and dynamic behaviour of the selected Nav1.7–ligand complexes over 250 ns. Overall, all systems exhibited an initial equilibration phase followed by varying degrees of structural stabilisation, indicating ligand-dependent effects on protein dynamics.

#### 2.2.1. Root-Mean Square Deviation

**Figure 4:**
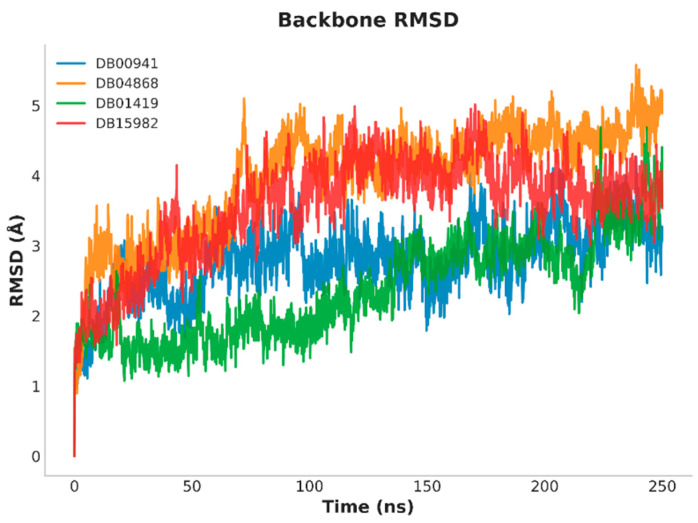
Backbone RMSD profiles of Nav1.7 in complex with selected compounds over 250 ns of molecular dynamics simulations, showing ligand-dependent stability and conformational deviations.

The backbone RMSD profiles (Figure 4) reveal distinct stability patterns among the complexes. DB01419 exhibits the lowest RMSD values throughout the simulation (~1.5–3.2 Å), indicating the most stable complex with limited structural deviation from the initial conformation. In contrast, DB04868 shows the highest RMSD values, reaching ~5.5 Å, suggesting increased conformational flexibility or reduced stabilisation within the binding site.

DB00941 maintains moderate stability, with RMSD values fluctuating around ~2.5–3.5 Å, indicating a relatively stable binding mode with manageable structural deviations. DB15982 displays intermediate behaviour, with RMSD values rising to ~4.0–4.5 Å before partially stabilising, reflecting a more dynamic interaction consistent with its diverse binding profile observed in docking. Overall, DB01419 appears to confer the greatest stabilising effect on the Nav1.7 structure, while DB04868 induces higher flexibility. These findings suggest that favourable docking scores do not necessarily translate into optimal dynamic stability, reinforcing the importance of MD simulations for candidate prioritisation [9,10].

**Figure 5:**
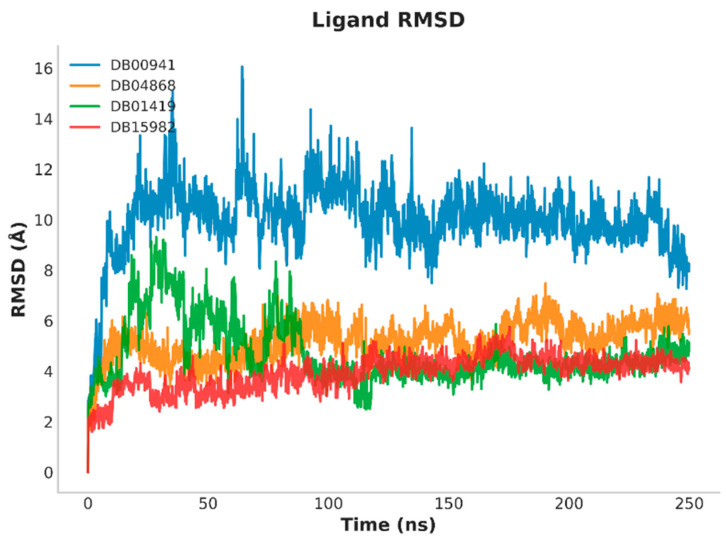
Ligand RMSD profiles of selected compounds in complex with Nav1.7 over 250 ns, highlighting differences in ligand stability and binding persistence within the pore.

Ligand RMSD profiles (Figure 5) reveal marked differences in binding stability across the four complexes. DB15982 and DB01419 maintain the lowest and most consistent RMSD values (~3–5 Å) throughout the simulation, reflecting stable positioning within the binding pocket with minimal conformational drift. DB04868 exhibits moderate fluctuations (~4–7 Å), consistent with limited local rearrangements while preserving overall binding site occupancy. DB00941, by contrast, displays substantially elevated and highly variable RMSD values (~8–12 Å), indicating that, in the protein-aligned reference frame, DB00941 has substantially displaced from its initial pose and largely drifted out of the central pore cavity rather than merely exhibiting local mobility. This dynamic instability is particularly striking given DB00941’s favourable static docking profile, underscoring the well-documented discordance between docking scores and true binding persistence.

Taken together, DB15982 and DB01419 emerge as the most dynamically stable binders, DB04868 occupies an intermediate position, and DB00941 demonstrates the poorest binding retention under physiological simulation conditions [10].

#### 2.2.2. Root-Mean Square Fluctuations

**Figure 6:**
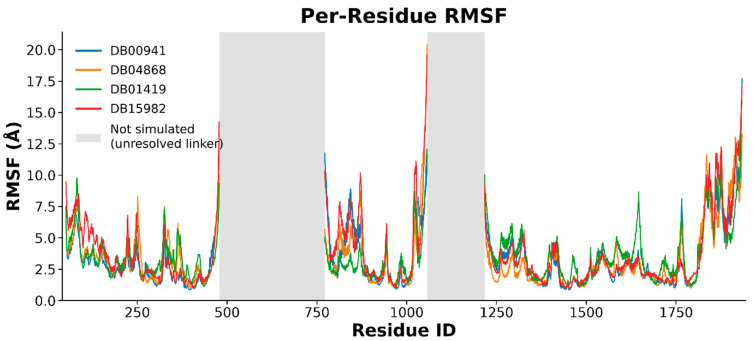
Per-residue RMSF of Nav1.7 in complex with selected compounds over 250 ns, highlighting residue-level flexibility and ligand-dependent stabilisation effects.

Per-residue RMSF profiles (Figure 6) show that Nav1.7 remains largely rigid across all four complexes, with fluctuations staying below ~3 Å throughout most of the sequence. As expected, elevated flexibility concentrates in loop regions and terminal segments, which lack the structural constraints of secondary structure elements. DB15982 is a clear outlier, with fluctuations peaking above 20 Å in discrete regions, pointing to genuine local destabilisation rather than global conformational change. DB01419 and DB04868 both suppress per-residue flexibility across the structure, with DB01419 yielding the consistently lower fluctuation profile of the two, evidence of a tighter, more stabilising interaction with the channel. DB00941 falls between these extremes, producing moderate fluctuations in several regions without the sharp peaks seen for DB15982. The shared flexibility peaks across all complexes reflect the intrinsic dynamics of the protein, whereas the ligand-dependent divergence in amplitude speaks to how differently each compound engages the pore-lining architecture. DB01419 confers the greatest structural rigidity, while DB15982 introduces the most pronounced local disorder.

#### 2.2.3. Principal Component Analysis

**Figure 7:**
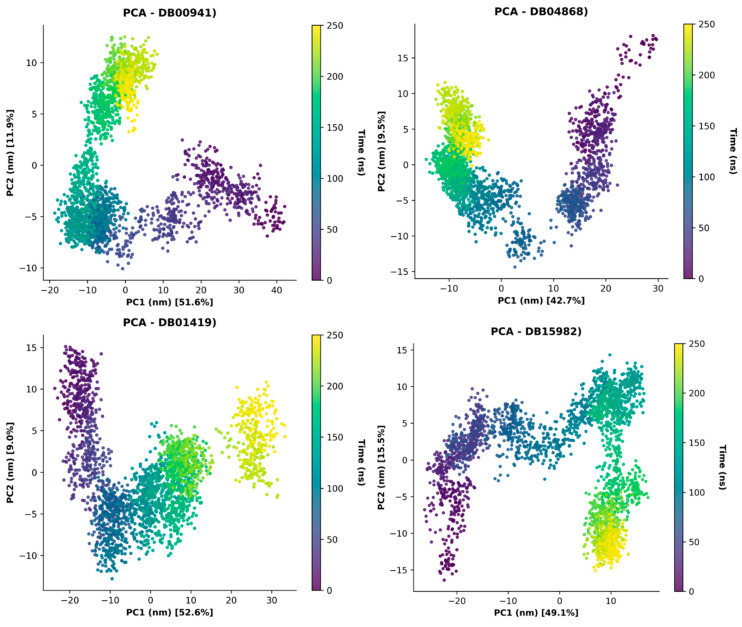
Principal component analysis of Nav1.7 complexes over 250 ns, showing ligand-dependent conformational sampling along the first two principal components (PC1 and PC2).

PCA was performed to characterise the dominant collective motions of Nav1.7 in each ligand-bound complex (Figure 7). The resulting conformational landscapes differ across systems; however, the projections of DB00941, DB04868, and DB15982 share a broadly similar curved topology, and these low-dimensional landscapes are therefore interpreted qualitatively rather than as definitive evidence that each compound imposes a mechanistically distinct dynamic signature. DB01419 produces a compact, well-defined distribution along the first two principal components, with trajectories transitioning gradually over time, a pattern consistent with restricted large-scale motion and stable sampling of a narrow conformational basin. DB15982 presents the opposite picture: broad dispersal across conformational space with sharp transitions between multiple well-separated clusters, reflecting a dynamic heterogeneity that mirrors the elevated RMSF peaks described above. DB04868 occupies a middle ground, with distinct conformational states and clear cluster separation, but with less continuous interconversion between them, suggesting periodic structural rearrangements rather than sustained flexibility. DB00941 shows tightly localised clusters with minimal overlap, indicative of transitions between discrete, stable-but-distinct conformations rather than fluid sampling, a pattern that, taken together with its elevated ligand RMSD, is consistent with a less stable binding mode. Across all four systems, the PCA results reinforce the hierarchy established by RMSD and RMSF analyses: DB01419 confines Nav1.7 to the most restricted conformational space, DB15982 induces the greatest structural heterogeneity, and DB04868 and DB00941 fall between these extremes for different mechanistic reasons. We emphasise that projections onto the first two principal components are inherently low-dimensional, basis-dependent, and sensitive to trajectory alignment and length; the qualitative differences described here are thus presented as descriptive of the sampled trajectories and not as quantitative mechanistic assignments.

#### 2.2.4. Protein–Ligand Contacts

**Figure 8:**
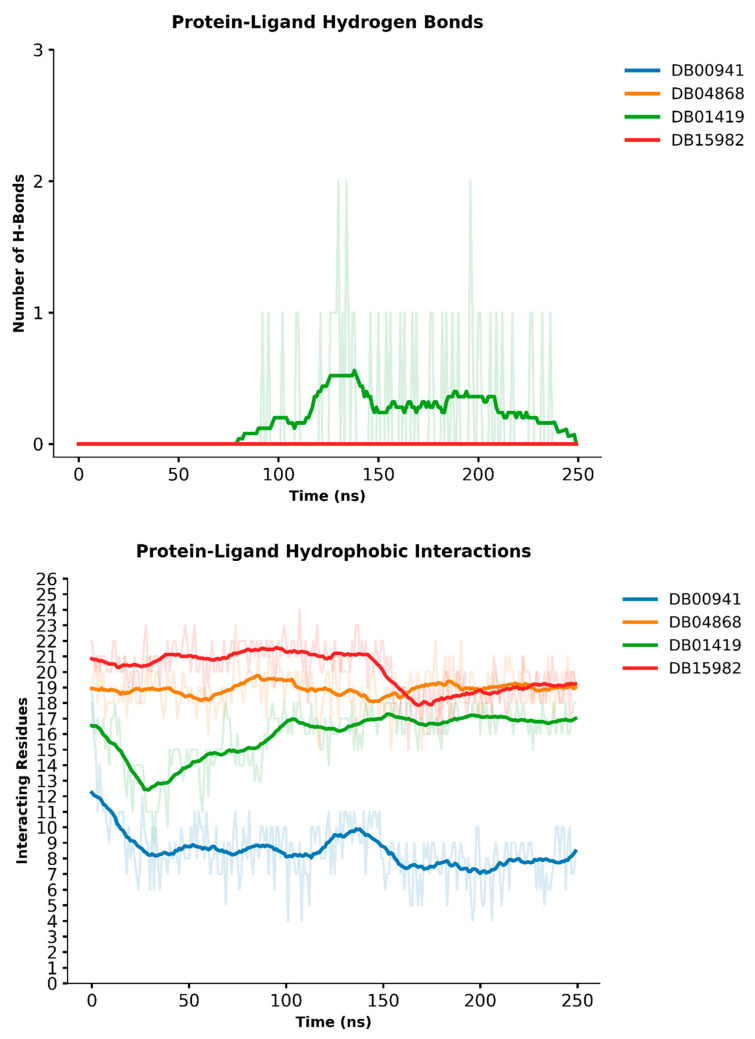
Time evolution of protein–ligand hydrophobic interactions and hydrogen bonds over 250 ns, illustrating the dominance of non-polar contacts and the limited, transient contribution of hydrogen bonding in stabilising Nav1.7–ligand complexes. Lighter, semi-transparent traces show the raw per-frame values, while the bold lines represent the running average over the trajectory.

Combined analysis of polar and non-polar contacts (Figure 8) reveals that binding within the Nav1.7 central pore is driven predominantly by hydrophobic interactions, with hydrogen bonding playing a minor, compound-specific role. Hydrophobic contacts remain stable throughout the simulation in all four complexes, though with clear quantitative differences: DB15982 and DB04868 engage the highest number of pore-lining residues, DB01419 maintains a somewhat smaller but consistent contact set, and DB00941 produces the weakest and most variable hydrophobic profile, a finding that aligns with its poor ligand RMSD and unstable PCA distribution. Hydrogen bonding is sparse across all systems. DB01419 is the sole compound to form intermittent hydrogen bonds, with low occupancy and transient peaks that nonetheless appear to contribute to the binding persistence and structural rigidity this compound confers on Nav1.7. The absence of any meaningful polar contacts for DB00941, DB04868, and DB15982 underscores that hydrophobic complementarity is both necessary and, for three of the four compounds, sufficient to maintain pore occupancy.

Taken together, the contact analysis frames the divergence in dynamic stability observed across the MD trajectories: the breadth and persistence of hydrophobic contacts set the baseline for binding, while the capacity to form even transient hydrogen bonds—as seen uniquely for DB01419, appears to confer an additional stabilising contribution that distinguishes it from the remaining candidates.

## 3. Discussion

The voltage-gated sodium channel Nav1.7 has emerged as one of the most compelling analgesic targets in modern drug discovery, owing to compelling human genetic evidence linking *SCN9A* gain-of-function mutations to severe pain syndromes such as inherited erythromelalgia and paroxysmal extreme pain disorder, and loss-of-function mutations to congenital insensitivity to pain [1,2]. This genetic validation, combined with the peripheral expression profile of Nav1.7 in dorsal root ganglion and sympathetic neurons, makes it an attractive target for non-opioid analgesics with potentially improved safety margins compared to non-selective sodium channel blockers [3,4]. Our study addresses the pressing need for novel Nav1.7-targeted candidates by repurposing FDA-approved compounds via structure-based virtual screening combined with molecular dynamics simulations.

The use of a high-resolution homology model derived from the 2.2 A cryo-EM structure (PDB: 7W9K) for virtual screening is well-supported by recent structural pharmacology studies demonstrating the accuracy of such models in guiding small-molecule binding predictions [14,15]. Targeting the central pore cavity of Nav1.7 is a well-established strategy, as this hydrophobic region accommodates pore-blocking drugs including local anesthetics, antiarrhythmics, and antiepileptic compounds [5,6]. Our docking results confirmed that binding within this cavity is overwhelmingly driven by hydrophobic interactions with conserved pore-lining residues such as Phe1503, Leu1010, and Ile1500. This is consistent with crystallographic and cryo-EM evidence showing that pore blockers, including XEN907, TC-N1752, and NaV1.7-IN2, engage the central cavity through van der Waals contacts with DIV-S6 and DII-S6 helices, with limited hydrogen bonding [6]. The high sequence conservation of this site across Nav isoforms, however, remains a recognised pharmacological challenge for achieving subtype selectivity [7].

The binding affinity range observed in our screen (−11.38 to −12.57 kcal/mol) is comparable to or exceeds values reported in previous AutoDock Vina-based screens targeting Nav1.7 and related ion channels [12,13]. DB04868 emerged as the top-ranked compound by docking score, consistent with its deeply buried, hydrophobic binding pose within the central cavity. However, MD simulations revealed that DB04868 produced the highest backbone RMSD values (~5.5 A), indicating substantial conformational flexibility of the Nav1.7-DB04868 complex under physiological conditions. This disconnect between docking score and dynamic stability is a well-documented limitation of static docking approaches and underscores the necessity of post-docking MD validation [9]. In contrast, DB01419 demonstrated the most stable dynamic behaviour across all four MD metrics: backbone RMSD, ligand RMSD, RMSF, and PCA, despite its lower docking score. This finding is consistent with reports showing that MD-derived stability metrics are more predictive of binding persistence than docking scores alone [10].

The interaction analysis further revealed that DB01419 is the only compound forming intermittent hydrogen bonds with Nav1.7, suggesting that even transient polar contacts can contribute meaningfully to binding stability and selectivity. This observation aligns with structure-guided studies of Nav1.7 antagonists showing that compounds capable of engaging both hydrophobic pore contacts and polar interactions with the selectivity filter residues tend to display superior binding profiles [14,15]. DB15982 showed intermediate ligand RMSD (~3–5 A) but exhibited pronounced per-residue RMSF peaks above 20 A in specific loop regions, reflecting a diverse but potentially destabilising interaction mode. DB00941, while interaction-rich in docking, displayed the highest ligand RMSD values (~8–12 A) and the weakest MD contacts, suggesting significant binding-pose rearrangement or partial displacement from the binding site over simulation timescales, which calls into question its suitability as a Nav1.7 inhibitor candidate under dynamic conditions.

The drug repurposing framework applied here is strategically advantageous. Screening FDA-approved compounds against Nav1.7 offers the benefit of pre-established safety profiles, pharmacokinetic data, and regulatory precedent, substantially reducing the time and cost of potential clinical translation [8]. Several FDA-approved drugs acting on sodium channels, including carbamazepine, lacosamide, and bupivacaine, have been shown to bind at the central pore or at nearby fenestrations of Nav1.7 [5], and the identification of additional approved compounds with pore-blocking potential could expand the therapeutic toolkit for neuropathic pain management. Nonetheless, the high sequence conservation of the pore domain across Nav subtypes means that selectivity profiling will be a critical next step for the identified candidates. Compounds showing Nav1.7 activity may also modulate cardiac Nav1.5 or CNS Nav1.2, necessitating electrophysiological characterisation. Because the pore-lining residues that dominate binding here (Phe1503, Leu1010, Ile1500, and Asn1504) are largely conserved across the Nav1.1-Nav1.9 family, any pose driven primarily by these contacts is expected a priori to translate to other isoforms; meaningful selectivity for pore binders typically arises instead from peripheral, isoform-divergent positions, for example within the domain-interface fenestrations and the periphery of the local-anaesthetic receptor site, rather than from the conserved core. The candidates nominated here should therefore be docked and simulated in parallel against Nav1.5 (cardiac), Nav1.2, and Nav1.6 (central nervous system) to quantify any selectivity window before experimental follow-up; such cross-isoform profiling was beyond the scope of the present pore-focused study and is identified as a key limitation below.

Several limitations of this study should be acknowledged. First, the use of a single rigid receptor conformation in docking does not account for induced-fit effects, which are known to influence Nav1.7 pore occupancy [15]. Relatedly, the docking protocol was not subjected to formal retrospective validation: redocking of the co-crystallised ligand to quantify pose-recovery RMSD, and enrichment or ROC analysis against a curated set of known Nav1.7 pore blockers and matched decoys, were not performed, so the screen is best interpreted as a hydrophobic-pore enrichment filter rather than a quantitatively validated affinity predictor. Second, while 250 ns MD simulations provide substantial conformational sampling, longer simulations or enhanced sampling methods such as replica-exchange MD could reveal slower conformational transitions relevant to drug binding and unbinding kinetics. We also did not compute time-resolved radius-of-gyration or binding-pocket-volume profiles, which would provide additional descriptors of system convergence that complement the RMSD and RMSF plateaus relied upon here; these are straightforward additions for future work. Relatedly, only a single 250 ns production replica was performed for each Nav1.7–ligand complex; multiple independent replicas would be required for rigorous statistical claims about ligand-induced stabilization, and the comparisons between compounds reported here should therefore be interpreted as descriptive of the trajectories sampled rather than as quantitative ranking under equilibrium conditions. Third, binding free energy calculations (e.g., MM-GBSA or alchemical perturbation methods) were not performed and would provide more quantitative affinity estimates to complement the docking and MD analyses. Fourth, the simulations were performed in an aqueous solvent box rather than in an explicit lipid bilayer; while this is reasonable for assessing ligand stability within a deeply buried, water-accessible pore cavity, an explicit POPC bilayer would provide a more faithful electrostatic environment for the membrane-embedded transmembrane domains and is the natural next step. More specifically, an explicit bilayer would impose lateral packing pressure on the S1–S6 transmembrane helices that is absent in an aqueous box, would modulate the local dielectric and the electrostatics at the lipid-facing fenestrations through which pore blockers are thought to partition, and could therefore influence channel conformation as well as the accessibility and residence time of ligands within the cavity; the comparatively high backbone RMSD (~5.5 A) of the least stable complex is consistent with partial relaxation of the unrestrained transmembrane region and should be re-evaluated in a membrane environment. Fifth, no cross-isoform docking or MD was performed against Nav1.5, Nav1.2, or Nav1.6, so the reported predictions cannot speak to subtype selectivity, which remains the dominant pharmacological challenge for pore-blocking Nav antagonists. Finally, the in silico predictions reported here require experimental validation through electrophysiological assays and binding studies to confirm Nav1.7 inhibitory activity and selectivity of the identified candidates.

Taken together, our results demonstrate that integrating structure-based virtual screening with multi-parameter MD analysis is a powerful strategy for prioritising FDA-approved candidates targeting Nav1.7. The divergence observed between docking rankings and MD-derived stability metrics reinforces the critical importance of dynamic simulations in computational drug discovery pipelines. DB01419 emerges as the most promising candidate based on its superior dynamic stability, while DB15982 warrants further investigation given its diverse interaction profile and stable ligand binding. These findings provide a foundation for subsequent experimental characterisation and rational optimisation of Nav1.7-targeted analgesics from the existing pharmacopoeia.

## 4. Materials and Methods

### 4.1. Protein Preparation

**Figure 9:**
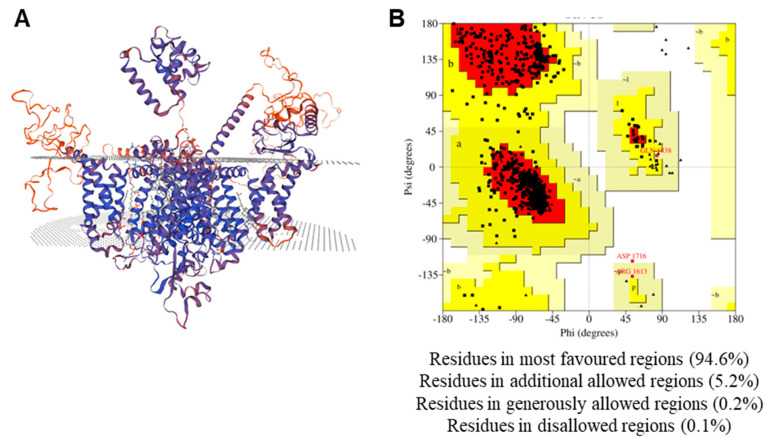
Structural modelling and validation of Nav1.7. (**A**) Homology model of Nav1.7, with reconstructed loop regions shown in orange. (**B**) Ramachandran plot indicating 94.6% of residues in the most favoured regions.

The Nav1.7 structure was initially retrieved from the RCSB PDB (PDB ID: 7W9K, resolution = 2.2 Å) [11]. However, this structure contains several missing loop regions. To obtain a complete model, the corresponding sequence was used for homology modelling with SWISS-MODEL [16]. As shown in Figure 9A, the resulting model exhibits high overall confidence, with lower confidence localized primarily to the reconstructed loop regions, which were absent in the experimental structure. Inclusion of these loops is necessary to ensure a structurally complete and physically consistent model suitable for molecular dynamics simulations. Figure 9B presents the Ramachandran plot, showing that 94.6% of residues fall within the most favoured regions, indicating good stereochemical quality of the modelled structure. Because the only modelled regions correspond to disordered or solvent-exposed loops absent in the cryo-EM map, and the central pore (the docking target in this study) is taken directly from the experimentally resolved coordinates, the homology-modelled regions do not contribute to the binding-site geometry used for screening; they are included solely to provide a continuous, physically reasonable structure for MD. In addition to these Ramachandran statistics, the SWISS-MODEL global model-quality estimates (QMEANDisCo and GMQE) generated during model building were inspected to confirm overall model reliability, and the reconstructed segments were verified to be confined to solvent-exposed cytosolic loops well removed from the central-pore docking site, so that any residual uncertainty in loop geometry does not propagate to the binding-site coordinates used for screening. The protein was subsequently prepared for docking using the Forli Lab’s Meeko package, version 0.7.0,to assign partial charges and generate the appropriate input format.

### 4.2. Ligand Library Preparation

A dataset of 12,171 compounds in 3D format was obtained from the DrugBank database [17]. An initial filtering step retained only FDA-approved drugs, yielding 2296 compounds for structure-based virtual screening. The compounds were energy-minimised using Open Babel [18] with the MMFF94 force field and subsequently converted to PDBQT format to assign atom types and partial charges for molecular docking.

### 4.3. Structure-Based Virtual Screening

Molecular docking was performed targeting the central pore of the Nav1.7 sodium channel. The co-crystallised ligand (9Z9) was used to define the binding site centre. A grid box centred at coordinates (209.50, 211.80, 192.46) with dimensions of 24 × 24 × 24 Å^3^ was employed to evaluate the binding affinity of FDA-approved compounds to the channel pore. Docking calculations were carried out using AutoDock Vina [12] with default parameters, and binding poses were ranked based on predicted binding affinity. Default exhaustiveness (8) and an energy_range of 3 kcal/mol were used; for each ligand the top-ranked pose by predicted binding affinity was retained for downstream analysis. AutoDock Vina v1.2.6 docking scores are inherently approximate and were used here only as an initial enrichment filter rather than as quantitative free-energy estimates; downstream prioritization was driven by interaction-fingerprint analysis and by the post-docking molecular dynamics metrics described in Section 4.4.

### 4.4. Molecular Dynamics Simulations

The top FDA-approved candidates identified from molecular docking were selected based on complementary criteria: DB04868 (anchor compound based on docking score), DB00941 and DB01419 (interaction-rich profiles), and DB15982 (diverse interaction pattern). The two higher-scoring compounds that were not advanced, DB08827 and DB01126, were de-prioritised because their top-ranked poses were dominated by a narrow, single-scaffold hydrophobic contact set and lacked the complementary polar or chemically diverse interaction fingerprints used here as selection criteria; this prioritisation strategy deliberately favoured interaction diversity and the capacity to form polar contacts over raw docking score, given the well-documented weak correlation between AutoDock Vina score and dynamic binding persistence. These compounds were subsequently subjected to molecular dynamics (MD) simulations to assess the stability and dynamic behaviour of their complexes with Nav1.7.

Protein–ligand systems were constructed using the CHARMM-GUI web interface [19], employing the CHARMM36m force field for the protein and appropriate parameters for the ligands [20]. Each complex was solvated in a rectangular TIP3P water box with a minimum padding distance of 10 Å from the solute to the box boundary. Sodium and chloride ions were added to neutralise the system and to achieve an ionic strength of 0.15 M. Ligand topologies and parameters were generated within the CHARMM-GUI Ligand Reader & Modeler module using the CHARMM General Force Field (CGenFF). For each compound, the top-ranked docked pose was used as the input three-dimensional conformation; hydrogen atoms were added consistent with the dominant protonation state expected at physiological pH (~7.4), and the automatically assigned atom types, bonded parameters, and partial charges were inspected, with the CGenFF penalty scores checked to confirm that no high-uncertainty by-analogy assignments were required. Each parameterised ligand was then combined with the prepared receptor prior to solvation.

Periodic boundary conditions were applied, and long-range electrostatics were treated using the Particle Mesh Ewald method with automatically generated FFT grid dimensions. Energy minimisation was performed to remove steric clashes and unfavourable contacts, followed by equilibration under constant volume (NVT) and constant pressure (NPT) conditions at 300 K and 1 bar, respectively. Temperature was maintained using a Langevin thermostat, while pressure was controlled using a Monte Carlo barostat.

Production MD simulations were then carried out for 250 ns using OpenMM, version 8.2.0 [21] with a 2 fs integration timestep using the LINCS-style constraint of bonds involving hydrogen atoms via SHAKE/HBonds in the OpenMM implementation, with trajectories saved at regular intervals for subsequent analysis. Post-simulation analyses, including root mean square deviation (RMSD), root mean square fluctuation (RMSF), principal component analysis (PCA), and ligand stability assessment, were performed using MDTraj [22] and MDAnalysis [23]. Unless otherwise stated, all reported deviations were computed after least-squares superposition of each trajectory frame onto the energy-minimised starting structure using the protein backbone (N, C-alpha, C) atoms as the fitting selection; backbone RMSD was then evaluated over these protein backbone atoms, whereas ligand RMSD was evaluated over ligand heavy atoms in this common, protein-aligned reference frame. Ligand RMSD therefore reports displacement of the ligand relative to the protein pore rather than internal conformational change of the ligand, and values approaching or exceeding ~8 A indicate that a compound has substantially departed from its initial pose within, or partially egressed from, the central cavity. A single 250 ns production replica was performed for each Nav1.7–ligand complex. We acknowledge that single-replica MD samples only a fraction of the accessible conformational landscape and is generally insufficient for rigorous statistical claims about stabilization or convergence; multiple independent replicas, longer aggregate sampling, and enhanced-sampling approaches (e.g., replica-exchange MD, Gaussian-accelerated MD, metadynamics) would be required to confirm convergence and to support quantitative thermodynamic claims. Convergence of RMSD/RMSF baselines and of the dominant principal-component basin during the latter half of each trajectory was used as an internal consistency check on the regime sampled. We also note that the present simulations were run in an aqueous box rather than an explicit lipid bilayer; while this is an acceptable simplification for assessing ligand stability within a deeply buried, water-accessible pore cavity, an explicit POPC bilayer would more faithfully model the membrane-embedded transmembrane domains and is a natural extension of this work.

## 5. Conclusions

This study presents an integrated workflow combining large-scale virtual screening of FDA-approved compounds against the Nav1.7 central pore with 250 ns post-docking molecular dynamics validation. From a library of 2296 FDA-approved drugs, four candidates, DB04868, DB00941, DB01419, and DB15982, were identified as having strong predicted affinity for the Nav1.7 pore, with binding affinities ranging from −11.38 to −12.57 kcal/mol and interaction profiles dominated by hydrophobic contacts with key pore-lining residues. Molecular dynamics simulations revealed that docking score alone is an unreliable predictor of binding stability: DB01419, despite ranking fourth by docking score, demonstrated the most stable complex formation across all MD metrics, including backbone RMSD, ligand RMSD, per-residue RMSF, and principal component analysis. Conversely, DB04868, which achieved the highest docking score, produced the most conformationally flexible complex under simulated physiological conditions. These findings highlight the value of MD simulations in post-docking candidate prioritisation. DB01419 and DB15982 are nominated as candidate scaffolds for downstream experimental evaluation as potential Nav1.7 modulators, providing a hypothesis-generating computational rationale for electrophysiological testing, selectivity profiling across Nav isoforms (most importantly Nav1.5 to assess cardiac safety, and Nav1.2/Nav1.6 to assess CNS safety), and potential lead optimisation toward non-opioid pain therapeutics.

## Data Availability

The original contributions presented in this study are included in the article. Further inquiries can be directed to the corresponding author.

## References

[1] Drenth J.P.H., Waxman S.G. (2007). Mutations in sodium-channel gene SCN9A cause a spectrum of human genetic pain disorders. J. Clin. Investig..

[2] Cox J.J., Reimann F., Nicholas A.K., Thornton G., Roberts E., Springell K., Karbani G., Jafri H., Mannan J., Raashid Y. (2006). An SCN9A channelopathy causes congenital inability to experience pain. Nature.

[3] Minett M.S., Nassar M.A., Clark A.K., Passmore G., Dickenson A.H., Wang F., Malcangio M., Wood J.N. (2012). Distinct Nav1.7-dependent pain sensations require different sets of sensory and sympathetic neurons. Nat. Commun..

[4] Shields S.D., Deng L., Reese R.M., Dourado M., Tao J., Foreman O., Chang J.H., Hackos D.H. (2018). Insensitivity to pain upon adult-onset deletion of Nav1.7 or its blockade with selective inhibitors. J. Neurosci..

[5] Nguyen P.T., Yarov-Yarovoy V. (2022). Towards structure-guided development of pain therapeutics targeting voltage-gated sodium channels. Front. Pharmacol..

[6] Zhang J., Shi Y., Huang Z., Li Y., Yang B., Gong J., Jiang D. (2022). Structural basis for NaV1.7 inhibition by pore blockers. Nat. Struct. Mol. Biol..

[7] Zhou G., Gao S., Wang L., Ma S., Ye H., Zhang Y., Xue W., Ouyang Q., Zhang L., Bhatt D.L. (2024). An artificial intelligence accelerated virtual screening platform for drug discovery. Nat. Commun..

[8] Pushpakom S., Iorio F., Eyers P.A., Escott K.J., Hopper S., Wells A., Doig A., Guilliams T., Latimer J., McNamee C. (2019). Drug repurposing: Progress, challenges and recommendations. Nat. Rev. Drug Discov..

[9] Shu J., Wang Y., Guo W., Chen H., Du Y., Liu H., Song J., Hu W., Cai S. (2024). Carbenoid-involved reactions integrated with scaffold-based screening generates a Nav1.7 inhibitor. Commun. Chem..

[10] Corry B. (2018). Physical basis of specificity and delayed binding of a subtype selective sodium channel inhibitor. Sci. Rep..

[11] Huang G., Liu D., Wang W., Wu Q., Chen J., Pan X., Shen H., Yan N. (2022). High-resolution structures of human Nav1.7 reveal gating modulation through α-π helical transition of S6IV. Cell Rep..

[12] Trott O., Olson A.J. (2010). AutoDock Vina: Improving the speed and accuracy of docking with a new scoring function, efficient optimization, and multithreading. J. Comput. Chem..

[13] Pan L., Bhatt D.L., Zhao Y., Zheng X., Zhao J., Pang X., Du G., Fan X., Tang J., Wang Y. (2020). In silico insight into voltage-gated sodium channel 1.7 inhibition for anti-pain drug discovery. Comput. Biol. Chem..

[14] Shen H., Liu D., Wu K., Lei J., Yan N. (2019). Structures of human Nav1.7 channel in complex with auxiliary subunits and animal toxins. Science.

[15] Wu Q., Huang J., Fan X., Wang K., Jin X., Huang G., Li J., Pan X., Yan N. (2023). Structural mapping of Nav1.7 antagonists. Nat. Commun..

[16] Waterhouse A., Bertoni M., Bienert S., Studer G., Tauriello G., Gumienny R., Heer F.T., de Beer T.A.P., Rempfer C., Bordoli L. (2018). SWISS-MODEL: Homology modelling of protein structures and complexes. Nucleic Acids Res..

[17] Wishart D.S., Feunang Y.D., Guo A.C., Lo E.J., Marcu A., Grant J.R., Sajed T., Johnson D., Li C., Sayeeda Z. (2018). DrugBank 5.0: A major update to the DrugBank database for. Nucleic Acids Res..

[18] O’Boyle N.M., Banck M., James C.A., Morley C., Vandermeersch T., Hutchison G.R. (2011). Open Babel: An open chemical toolbox. J. Cheminform..

[19] Jo S., Kim T., Iyer V.G., Im W. (2008). CHARMM-GUI: A web-based graphical user interface for CHARMM. J. Comput. Chem..

[20] Huang J., Rauscher S., Nawrocki G., Ran T., Feig M., de Groot B.L., Grubmüller H., MacKerell A.D. (2017). CHARMM36m: An improved force field for folded and intrinsically disordered proteins. Nat. Methods.

[21] Eastman P., Swails J., Chodera J.D., McGibbon R.T., Zhao Y., Beauchamp K.A., Wang L.-P., Simmonett A.C., Harrigan M.P., Stern C.D. (2017). OpenMM 7: Rapid development of high performance algorithms for molecular dynamics. PLoS Comput. Biol..

[22] McGibbon R.T., Beauchamp K.A., Harrigan M.P., Klein C., Swails J.M., Hernández C.X., Schwantes C.R., Wang L.-P., Lane T.J., Pande V.S. (2015). MDTraj: A modern open library for the analysis of molecular dynamics trajectories. Biophys. J..

[23] Michaud-Agrawal N., Denning E.J., Woolf T.B., Beckstein O. (2011). MDAnalysis: A toolkit for the analysis of molecular dynamics simulations. J. Comput. Chem..

